# Optional Web-Based Videoconferencing Added to Office-Based Care for Women Receiving Psychotherapy During the Postpartum Period: Pilot Randomized Controlled Trial

**DOI:** 10.2196/13172

**Published:** 2019-05-20

**Authors:** Rebecca Yang, Simone N Vigod, Jennifer M Hensel

**Affiliations:** 1 Women's College Hospital Institute for Health System Solutions and Virtual Care Women's College Hospital Toronto, ON Canada; 2 Department of Psychiatry Women's College Hospital Toronto, ON Canada; 3 Women's College Research Institute Women's College Hospital Toronto, ON Canada; 4 Institute for Health Policy, Management, and Evaluation University of Toronto Toronto, ON Canada; 5 Department of Psychiatry University of Manitoba Winnipeg, MB Canada

**Keywords:** mental health, psychotherapy, postpartum period, videoconferencing

## Abstract

**Background:**

Depression and anxiety during the postpartum period are common, with psychotherapy often being the preferred method of treatment. However, psychological, physical, and social barriers prevent women from receiving appropriate and timely psychotherapy. The option of receiving psychotherapy through videoconferencing (VC) during the postpartum period presents an opportunity for more accessible and flexible care.

**Objective:**

The aim of this study was to assess the feasibility, acceptability, and preliminary effectiveness of optional VC added to usual office-based psychotherapy, with a psychotherapist during the postpartum period.

**Methods:**

We conducted a pilot randomized controlled trial with 1:1 randomization to office-based care (treatment as usual; TAU) or office-based care with the option of VC (treatment as usual plus videoconferencing; TAU-VC) for psychotherapy during the postpartum period. We assessed the ability to recruit and retain postpartum women into the study from an urban perinatal mental health program offering postpartum psychotherapy, and we evaluated the uptake, acceptability, and satisfaction with VC as an addition to in-person psychotherapy. We also compared therapy attendance using therapist logs and symptoms between treatment groups. Symptoms were assessed at baseline and 3 months postrandomization with the Edinburgh Postnatal Depression Scale, Generalized Anxiety Disorder 7-item, and Parental Stress Scale. Furthermore, 3-month scores were compared between groups with intention-to-treat linear mixed-effects models controlling for baseline score.

**Results:**

We enrolled 38 participants into the study, with 19 participants in each treatment group. Attendance data were available for all participants, with follow-up symptom measures available for 25 out of 38 participants (66%). Among the 19 TAU-VC participants, 14 participants (74%) utilized VC at least once. Most participants were highly satisfied with the VC option, and they reported average savings of Can $26 and 2.5 hours in travel and childcare expenses and time per appointment. There were no significant differences between the 2 groups for psychotherapy attendance or symptoms.

**Conclusions:**

The option of VC appears to be an acceptable method of receiving psychotherapy for postpartum women, with benefits described in costs and time savings. On the basis of this small pilot sample, there were no significant differences in outcomes between office-based care with or without the option of VC. This study has demonstrated the feasibility of such a program in an urban center, which suggests that a larger study would be beneficial to provide evidence that is more conclusive.

## Introduction

The need for high-quality, accessible mental health care is significant, with important individual and societal implications. However, the lack of services, underawareness of services, and stigma and attitudes associated with mental health are large barriers to help seeking [1], with at least 50% of those affected not receiving adequate care [2]. The postpartum period can be a particularly vulnerable time for women requiring mental health support. Up to 15% of women experience postpartum mood disorders [3,4], with most preferring psychotherapy over medication for the treatment of their mental illnesses [5]. However, childcare responsibilities and physical recovery, particularly after a surgical birth, create barriers for regular therapy attendance [6-8]. Barriers to care often lead to delayed care, treatment discontinuation, and the lack of follow-up on recommendations. Untreated symptoms of perinatal mental illness can last for months or years, leading to persistent mental health problems and decreased quality of life for both the woman and child [9,10].

Virtual care tools, which facilitate remote interactions between patients and health care providers through information technology [11], have gained traction as potential solutions to deliver effective treatment for most mental health needs [12-14]. They can address many barriers associated with in-person care, such as travel distance and time, travel costs, and stigma [5,15]. In addition, patients may be more motivated to seek and continue treatment if they are in a familiar environment and can avoid potentially stressful situations, such as driving in urban cities and navigating a hospital [16]. More women are increasingly accessing the internet for support and information during the postpartum period [17]. A study by Maloni and colleagues [6] found that among postpartum women with depression and women who experienced pregnancy complications, over 90% of the women demonstrated an interest in internet-based interventions, with 40% indicating a specific interest in chatting virtually with a provider with expertise in postpartum depression. Similarly, Sawyer et al found that access to a nurse and peers through an internet-based chat forum yielded improved parental stress scores compared with standard treatment alone [18]. A survey conducted in our own ambulatory outpatient mental health program, which has a special focus on perinatal mental health, found that pregnancy factors and/or childcare were cited as a common barrier to office-based care [19]. Among all survey respondents with an identified barrier, 93% of these patients were interested in internet-based treatment tools, and 76% of these patients were specifically interested in videoconferencing (VC) to receive their care from home, using a personal device [19]. Studies on VC-based psychotherapy have demonstrated similar treatment outcomes when directly compared with the face-to-face option [16,20].

To our knowledge, no studies have specifically assessed the option of VC in addition to office-based care to determine if it added any benefits. By overcoming the barriers to care that can be present in varying ways across a course of treatment, the addition of VC to an office-based treatment program could provide a very patient-centered option to receive care. This could lead to improved therapy adherence and completion rates and possibly better outcomes as a result. In this study, we assessed the acceptability, uptake, and preliminary effectiveness of adding the option to receive psychotherapy delivered by VC to standard office-based psychotherapy for women experiencing mood and anxiety problems during their first year postpartum.

## Methods

### Study Design

This study was a pilot randomized controlled trial, comparing usual office-based care (treatment as usual; TAU) with office-based care with the option of VC on a personal device (treatment as usual plus videoconferencing; TAU-VC) for psychotherapy for mood or anxiety problems in the postpartum period. Participants were randomized 1:1 into 1 of the 2 treatment arms, and outcomes were measured at baseline and 3 months postrandomization. This study had 3 objectives: (1) assess the ability to recruit and retain postpartum women into the study, (2) evaluate the uptake and satisfaction with VC as an addition to in-person psychotherapy, and (3) compare therapy attendance and symptoms after 3 months between those receiving the option of VC with those receiving usual care. Research ethics approval was obtained from the Women’s College Hospital Research Ethics Board.

### Study Setting

Participants were recruited from a specialized mental health program in an ambulatory hospital within an urban city in Ontario, Canada, which provides publicly funded individual psychotherapy for mood, anxiety, obsessive-compulsive, or trauma or stressed-related disorders in pregnancy and up to 1 year postpartum delivered by highly trained psychotherapists with a master’s degree in social work. The program accepts referrals from primary care, midwifery, obstetricians, and psychiatrists from across Ontario, although the vast majority of women who receive care reside in the greater Toronto region (population ~6.4 million). The program receives approximately 1200 referrals annually, and it will provide assessment and treatment to women within the first year postpartum. The clinic does provide prebooked childcare during daytime hours. Individuals referred to the program are assessed by a psychiatrist, and if deemed suitable for psychotherapy, they may be referred to group or individual services. A psychotherapist contacts referred individuals to schedule an initial appointment, usually within 1 month of their psychiatric assessment. Therapists receive about 10 referrals for individual treatment a month, of which, approximately 70% convert into a course of treatment.

### Recruitment

Study recruitment took place from June 2016 to July 2017. We aimed to recruit 40 participants, as has been recommended for pilot studies of an intervention where the goal is to have sufficient variability to examine processes in the implementation of the protocol [21]. Participation was initially just offered to only new referrals to the clinic, but it was expanded to include patients already in treatment after trial commencement to increase recruitment rate. Psychotherapists introduced the study to newly referred patients during the initial call about treatment and to existing patients during a therapy session. The inclusion criteria for this study were patients in the program, aged 18 years or older, referred to psychotherapy for mood and/or anxiety symptoms, who had access to and ability to use a Web-enabled personal device or computer with the required audiovisual communication capability, and had a functioning email address (a requirement for the VC platform). Patients could be approached when pregnant if they intended to continue or start psychotherapy postpartum, but the study protocol was not initiated until after the woman returned for therapy postpartum. For postpartum patients, only women less than 9 months postpartum were included, to allow a minimum of 3 months of treatment before they reached 1 year postpartum and were discharged from the program. Patients with acute mania or psychosis or severe suicidal ideation with planning and intent were excluded. Therapists provided the contact information of interested patients to a research assistant. The research assistant contacted interested patients to introduce and explain the study. If the patient was still interested, the research assistant obtained and documented informed verbal consent over the phone and emailed a copy of the completed consent form to the patient.

### Intervention Groups

#### Treatment as Usual

In the TAU group, participants received the standard form of psychotherapy available in the clinic. In the program, psychotherapists provide first-line evidence-based treatments for depression and anxiety, including cognitive behavior therapy and interpersonal therapy, adapted for the postpartum context. This treatment is mostly provided in person, although sessions are sometimes delivered by telephone on an ad hoc basis if a patient is unable to attend in person because of medical or last-minute childcare problems. Patients may also receive psychiatric care, including medication management, from the program psychiatrist until approximately 1 year postpartum. At this point, patients are referred back to their primary care provider for ongoing management.

#### Treatment as Usual Plus Videoconferencing

In the TAU-VC group, participants had access to TAU with the added option of having any of their treatment sessions over VC. The VC platform was hosted by the Ontario Telemedicine Network, a government-funded organization that supplies telemedicine services free of charge to health care organizations in Ontario. The secure platform is available across the province and can be used for communication among health professionals, between patients and health professionals, or as a webcast platform. To access the Web-based VC platform, participants had to download a plug-in for their computer, which required a webcam and microphone or an app for their mobile phone or tablet. All the therapists had unique accounts to log into the portal on their office computers. Therapists were able to schedule a therapy session and invite the participants by emailing them a personal link that included the date and time. Participants could attend sessions from their desired location, but they were encouraged to ensure the location was private enough so that they could fully participate in the session. When the appointment time arrived, participants and therapists could enter the virtual session. In addition to video and audio sharing, therapists had the ability to share their screens, which allowed them to show worksheets or other visual materials during the session. All participants who received access to VC received an instruction pamphlet by email and had a brief test call with the research assistant to ensure their device met the required technical specifications, to give a demonstration of the platform, and to troubleshoot any initial technical issues. During the study, participants in the TAU-VC group were encouraged, but not required, to use VC for their therapy sessions, and they were still able to access in-person therapy sessions and phone support as per standard care. Participants were also informed that therapists could, at their discretion, recommend an in-person visit if they felt it was warranted for clinical reasons. Technical support could be obtained from the research assistant or from the Ontario Telemedicine Network. All program psychotherapists (3 individuals) provided VC sessions during the study.

### Randomization and Blinding

Participants were allocated 1:1 to the intervention and control groups using simple randomization. For randomization, 20 slips of paper labeled with *TAU* and 20 with *TAU-VC* were placed in opaque envelopes by a research staff member who was not involved with this study. Upon documentation of informed consent, the research assistant opened 1 of the envelopes, revealed the group allocation, and communicated it to the participant and psychotherapist. If a participant withdrew before being informed of the participant’s allocation, the paper was returned to an envelope and put back in the pile. Participants and therapists were unblinded; data analyses were blinded to group allocation.

### Data Collection

Baseline data were collected for all participants by the research assistant. If the participant was postpartum, data were collected at the time of recruitment, and if the participant was pregnant when recruited, data were collected following delivery of the child and upon reengaging in therapy. Baseline data included participant age, age of child or children, childcare support, and exposure to previous therapy, which referred to whether the participant had any previous therapy experience in her lifetime. Additional data, including address (to calculate travel distance from the clinic) and whether the participant was a newly referred or existing patient at the time of recruitment, were provided by therapists or extracted from individual clinical charts.

Baseline symptom and function measures were administered by a Web-based survey. Participants provided an email address, and they were sent a link to a survey hosted on FluidSurveys, which was later transitioned to SurveyMonkey after an institutional change in survey host. All participants received another survey link by email as a reminder to complete follow-up measures 2 weeks before reaching 3 months, and they received the link again at 3 months if the survey had not been completed. A follow-up phone call was made to any outstanding surveys at 2 weeks after the 3-month time point. At follow-up, participants in the TAU-VC group were asked additional questions about their use of and satisfaction with VC.

The psychotherapists completed a therapy log for each of their participants, which documented the length of time (in minutes) and format (in person, VC, or telephone) of each session, along with cancellations and no-shows and how often a child was present for the session.

### Measures

#### Edinburgh Postnatal Depression Scale

The Edinburgh Postnatal Depression Scale (EPDS) is a self-report depression screening measure that has been validated for use in pregnancy [22]. EPDS scores >12 are predictive of a diagnosis of major depressive disorder. The EPDS is able to detect women with depression in the perinatal period better than traditional depression measures because of the increased weight given to anxiety symptoms that appear to be more common in perinatal than in nonperinatal depression [22].

#### Generalized Anxiety Disorder 7-Item

The Generalized Anxiety Disorder 7-item (GAD-7) assesses symptoms of general anxiety. It has 7 items rated on a 4-point scale from “never” to “nearly every day,” as well as 1 perceived impairment rating. A score of ≥10 is highly suggestive of a problem with anxiety. A reduction of 5 points corresponds to a clinically meaningful improvement, with a reduction in score of 50% representing response [23].

#### Parental Stress Scale

The Parental Stress Scale (PSS) is an 18-item questionnaire that was developed to measure the level of stress associated with raising children. All items are scored on a 5-point Likert scale from “strongly disagree” to “strongly agree.” The questionnaire has shown good reliability and internal consistency [24]. It has been used to study parenting stress in a variety of parent-child circumstances, and it is correlated with measures of depression [25]. A total score is obtained by summing all items.

#### Telemedicine Satisfaction Questionnaire

The Telemedicine Satisfaction Questionnaire (TSQ) was originally developed and studied by Yip et al [26] to assess satisfaction with telemedicine. It has 15 items rated on a 5-point Likert scale from “strongly disagree” to “strongly agree.” The authors reported good internal validity and consistency. A factor analysis yielded 3 components: quality of care provided [8 items], similarity to in-person face-to-face interaction (5 items), and perception of the interaction (1 item). This study used the original TSQ with the term “telemedicine” substituted with “video visits.” An overall score can be created by summing all items, or subscale scores can be calculated for the component domains. Individual items may also be examined to assess the positive and negative aspects of the intervention [26]. In this study, 2 additional items were added: “I am going to miss the equipment when the project ends,” and “I would be willing to pay for video visits privately.” These latter questions were added to gather additional data regarding interest in the intervention.

#### Patient Reported Costs Questionnaire

The Patient Reported Costs Questionnaire (PRCQ) was developed by the research team to assess the participant costs and cost savings associated with access to VC. Respondents were asked to estimate the amount and source of time and money they saved when they attended a session of therapy with VC compared with in person.

### Data Analysis

#### Objective 1: Recruitment and Retention

We documented the number and rate of referrals, baseline demographics and symptoms scores, and the proportion of participants who provided follow-up data at the outcome time points.

#### Objective 2: Uptake and Satisfaction With Videoconferencing

Descriptive statistics were generated to examine uptake of the intervention as defined as number and percentage of sessions attended by VC. TSQ and PRCQ items were analyzed descriptively.

#### Objective 3: Comparison of Therapy Attendance and Symptoms

Session attendance variables were summarized as group means or medians and compared between groups with unpaired 2-tailed *t* tests or nonparametric tests where medians were reported. Endpoint EPDS, GAD-7, and PSS total scores at 3 months were separately compared between groups with intention-to-treat linear mixed-effects models, including the relevant baseline score as a covariate. We conducted the same analyses in the subgroup of participants with baseline EPDS scores greater than 12 to examine the participants with baseline symptoms suggestive of a major depressive disorder [22].

## Results

### Objective 1: Recruitment and Retention

**Figure 1:**
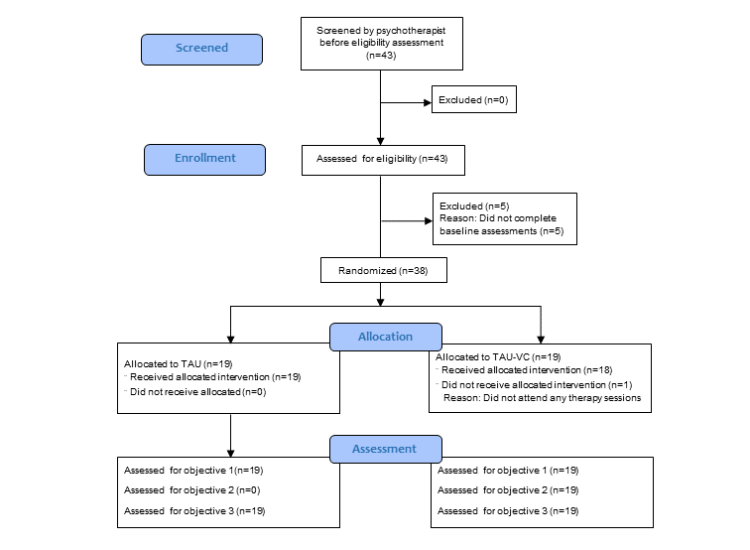
Participant flow for the pilot randomized controlled trial. Refer to Study Design for the study objectives. TAU: treatment as usual; TAU-VC: treatment as usual plus videoconferencing.

Participant flow through the study is outlined in Figure 1. Between June 2016 and July 2017, a total of 43 individuals were referred to the study. Although all the participants provided consent to participate, 5 did not complete baseline measures; therefore, they were not allocated to a treatment condition. A total of 38 participants were therefore included in the study, 19 in each group. At 3 months postrandomization, therapy attendance data were available for all participants (n=38/38, 100%), and 25 out of 38 (66%) participants completed symptom scale scores, with a slightly higher completion rate in the TAU-VC (n=14/38, 78%) than the TAU (n=11/38, 61%) condition.

Table 1 summarizes the baseline data for individuals randomized by treatment group. The average baseline EPDS, GAD-7, and PSS scores in both groups indicate moderate postnatal depression, generalized anxiety, and parental stress, respectively.

**Table 1:** Baseline characteristics of participants.

Variable	TAU-VC^a^ (n=19)	TAU^b^ (n=19)
Age (years), mean (SD)	33.8 (3)	33.8 (3)
Distance from clinic (in km), mean (SD)	7.8 (6)	7.7 (4)
Married or common-law, n (%)	18 (95)	16 (84)
Single, n (%)	1 (5)	3 (16)
Family or formal childcare support, n (%)	10 (53)	15 (79)
Index child age at start of study in weeks, median (IQR^c^)	8.0 (14)	9.0 (21)
Any other children, n (%)	10 (49)	10 (51)
Previous therapy experience, n (%)	16 (89)	15 (79)
New patient, n (%)	9 (47)	6 (32)
Existing patient, n (%)	10 (53)	13 (68)
Baseline Edinburgh Postnatal Depression Scale, mean (SD)	13.3 (6)	13.6 (5)
Baseline Generalized Anxiety Disorder 7-item, mean (SD)	10.5 (5)	9.0 (5)
Baseline Parental Stress Scale, mean (SD)	45.3 (12)	49.5 (12)

^a^TAU-VC: treatment as usual plus videoconferencing.

^b^TAU: treatment as usual.

^c^IQR: interquartile range.

### Objective 2: Uptake and Satisfaction With Videoconferencing

**Figure 2:**
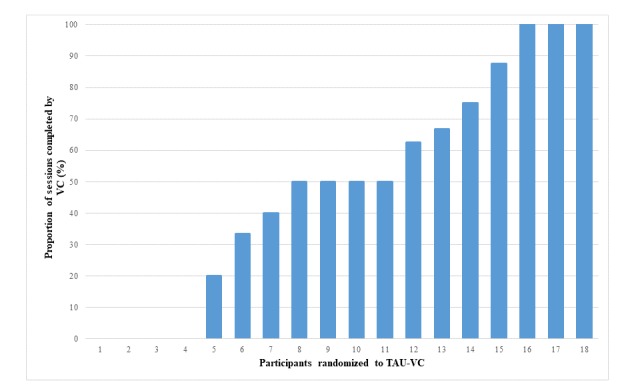
Proportion of sessions completed by videoconferencing among participants who were randomized to treatment as usual plus videoconferencing group. TAU-VC: treatment as usual plus videoconferencing; VC: videoconferencing.

VC was used at least once by 14 (74%) of the participants in the TAU-VC group. The median number of VC sessions was 2 (range: 0-8). On average, users attended 50% of their sessions with VC, with 4 participants using VC for 100% of their therapy sessions and 4 participants using it for none of their sessions. Figure 2 displays the proportion of sessions completed by VC, phone, and in person among participants in the TAU-VC group. Although 19 participants were allocated to the TAU-VC condition, 1 participant did not attend any sessions of any kind and is not shown in the figure.

A total of 11 of the 14 VC users completed the TSQ. Of them, 9 VC users (82%) agreed or strongly agreed that they felt VC allowed them to attend therapy sessions more frequently. In a subgroup comparison between TAU-VC participants who were TSQ responders and TAU participants, TSQ responders attended more sessions face to face (4.6 vs 2.9, *P*=.06), with total therapist contacts being more similar (5.1 vs 4.8, *P*=.80), indicating that the TAU groups had a higher number of phone contacts. The average TSQ item score was 4.7 out of 5, with average scores of 4.7 (SD 0.43), 4.7 (SD 0.31), and 4.6 (SD 0.67) in the domains of quality of care provided, similarity to in-person face-to-face interaction, and perception of the interaction, respectively. All participants said they would miss having access to VC when the project ended, but only 1 participant expressed a high willingness to pay out of pocket to have it as an available option.

All participants who used VC at least once reported saving money and time when attending therapy sessions with VC compared with in person. Specifically, the average cost savings was Can $26 (SD 19.7, range: Can $7-$70) in travel, childcare, and other expenses per session, and the average time savings was 2.5 hours (SD 1.6, range: 1-7 hours) in preparation and transportation per session. There was a small start-up cost to purchase the webcams for the therapist computers (approximately Can $200 per therapist). There were no operating costs for the VC platform, as it was provided courtesy of a government-funded organization in Ontario.

### Objective 3: Comparison of Therapy Attendance and Symptoms

There were no significant differences between the 2 groups for the total number of sessions attended, the average length of time per session, the number of no-shows or cancellations, and the number of sessions with a child in attendance (Table 2). Participants in the TAU-VC group tended to attend more face-to-face sessions (in person or by VC) and fewer phone sessions than the TAU participants, but the differences did not reach statistical significance.

**Table 2:** Summary of therapy attendance variables between groups.

Variable^a^	TAU-VC^b^ (n=19)	TAU^c^ (n=19)	*P* value
Total number of sessions (all types), mean (SD)	4.5 (2.1)	4.8 (2.9)	.71
Total time (min)/session, median (IQR^d^)	55.7 (10.0)	60.0 (12.5)	.58
Cancellations/no-shows, median (IQR)	1.0 (2.0)	1.0 (2.0)	.86
Total number of face-to-face sessions (at clinic or VC), mean (SD)	3.8 (2.2)	2.9 (2.4)	.24
Total phone sessions, median (IQR)	0.0 (1.0)	1.0 (2.0)	.18
Sessions with child attending, median (IQR)	0.0 (3.0)	0.0 (2.0)	.71

^a^Continuous variables presented as mean (SD) are compared with 2-tailed unpaired *t* tests; variables presented as median (IQR) are compared with Mann-Whitney *U* tests.

^b^TAU-VC: treatment as usual plus videoconferencing.

^c^TAU: treatment as usual.

^d^IQR: interquartile range.

**Table 3:** Estimated marginal means and treatment effect size for all outcomes based on linear mixed models, adjusted for baseline scores.

Outcome	Estimated marginal 3-month total score, mean (SD)		*F* test (*df*)	Treatment effect size (95% CI)
	TAU-VC^a^	TAU^b^		
Edinburgh Postnatal Depression Scale	11.7 (4.5)	12.1 (4.5)	.053 (22)	–0.42 (–4.23 to 3.91)
Generalized Anxiety Disorder 7-item	8.7 (4.5)	9.1 (4.6)	.050 (21)	–0.44 (–4.49 to 3.62)
Parental Stress Scale	47.6 (6.2)	44.2 (6.2)	1.89 (22)	3.42 (–1.74 to 8.59)

^a^TAU-VC: treatment as usual plus videoconferencing.

^b^TAU: treatment as usual.

Similarly, there were no significant differences in 3-month EPDS, GAD-7, or PSS between groups (see Table 3). When analyses were restricted to those with baseline EPDS >12 (n=10 in the TAU-VC group and n=9 in the TAU group), there were again no significant differences between groups for 3-month EPDS (*F*_1,9_=0.058, *P*=.81), GAD-7 (*F*_1,9_=0.004, *P*=.95), or PSS (*F*_1,9_=0.003, *P*=.96).

## Discussion

### Principal Findings

In this pilot study, we demonstrated feasibility of recruitment and retention of participants from a program providing psychotherapy to postpartum women with mood and/or anxiety symptoms into a study offering VC as an adjunct to office-based care. We also showed that participants used the intervention, and they reported high satisfaction and substantial savings in time and money. We found no statistically significant differences in total number of sessions attended, and although the number of participants was small, there was no evidence that symptom outcomes differed between those with the option of VC compared with those attending office-based appointments only. Uptake of VC in the intervention group was quite variable. In this study, we offered VC as an option to attending in-person sessions at the clinic, and 74% of the participants used it at least once. Participants used VC for 50% of the sessions on average, with some participants using it often and others choosing not to use it at all. Variability in the uptake of virtual care tools is commonly observed, and it may be attributed to differences in patient-perceived barriers or preferences [27,28]. Among those who used VC, overall, participants were satisfied with the technology. High scores were provided across all the domains of the TSQ, including quality of care provided, similarity to in-person face-to-face interaction, and perception of the interaction. This finding is consistent with other studies that demonstrate high levels of patient acceptance and satisfaction with virtual mental health care [29,30], even when they experience frustration because of technical difficulties [16]. Participants’ satisfaction may be associated with their ability to choose the format to receive psychotherapy week to week, increasing their perception of autonomy [31]. Participants subjectively felt that access to optional VC allowed them to attend therapy sessions more frequently. We would hypothesize that by overcoming barriers to care, VC would increase attendance. However, this finding was not statistically observed between groups. A larger study would be better able to determine if access to optional VC affects overall therapy attendance or if access to optional VC affects therapy attendance for certain subgroups.

There appeared to be other advantages of VC as perceived by our participants. Those who used VC reported an average cost savings of Can $26 and time savings of 2.5 hours per session. Over numerous sessions, characteristic of a course of psychotherapy, this amount would add up to significant savings in time and money. To our knowledge, this is the first study to explore patient-rated costs for the use of VC in psychotherapy. Although participants were satisfied with VC, only 1 participant indicated a willingness to pay for VC access out of pocket if it were not funded by the hospital. This may not be surprising, given that our program exists in a government-funded health care system where patients do not normally have to pay out of pocket for any ambulatory care programs situated in a hospital. Although the sample size was small, it was reassuring that participants who used VC had similar outcomes to those who received TAU. Sometimes, when technology is leveraged, the increased convenience and ease of access have to be weighed against a possible reduction in treatment efficacy [32]. Although a larger study would be required to generate more certainty about the outcomes, it is promising that it does not appear to be the case for a mental health treatment model, where in-person care is supplemented by virtual care for postpartum women.

### Limitations

This pilot study used a sample of 38 participants recruited from an academic ambulatory hospital in an urban city; therefore, its patients may have access to more resources than other parts of the province where access barriers are more pronounced. Our program is unique in that it offers specialized care in an ambulatory hospital that provides access to free childcare during appointments, which eliminates some of the barriers to care in the postpartum population. In addition, we have access to a secure government-funded VC platform. In this setting, we evaluated the option of VC for treatment, which in some areas may not be feasible when there is no access to office-based treatment and VC is the only option. Over 80% of the participants in the study had had some therapy experience before being referred to our program; therefore, the results may not be as generalizable to a therapy-naïve cohort. That said, we demonstrated the feasibility of recruiting and retaining postpartum women receiving psychotherapy for mood and anxiety difficulties to receive the option of VC in addition to office-based care. In addition, acceptability of the intervention was high, and there were no indications of significant differences in outcomes, supporting that a larger study is warranted.

### Conclusions

This novel research protocol explored the acceptability and preliminary effectiveness of VC in combination with a treatment-as-usual protocol of in-person and telephone-based care for psychotherapy during the postpartum period within an urban center. VC was perceived as an acceptable format in which to receive psychotherapy, with patient-reported cost and time savings. Virtually available options to help patients overcome barriers to care can enhance the patient-centeredness of ambulatory care, most likely without any compromise to patient outcomes in this population. The results herein support proceeding to a larger study to definitively evaluate this protocol for its efficacy in this vulnerable population. Future work should also evaluate the use of VC in other urban settings and patient populations.

## Abbreviations

EPDS: Edinburgh Postnatal Depression Scale
GAD-7: Generalized Anxiety Disorder 7-item
PRCQ: Patient Reported Costs Questionnaire
PSS: Parental Stress Scale
TAU: treatment as usual
TAU-VC: treatment as usual plus videoconferencing
TSQ: Telemedicine Satisfaction Questionnaire
VC: videoconferencing

## Appendix

Multimedia Appendix 1 CONSORT-EHEALTH checklist (V 1.6.1).
